# Comparative Transcriptome Analysis of the Effects of a Non-Insect Artificial Diet on the Nutritional Development of *Harmonia axyridis*

**DOI:** 10.3390/insects16040380

**Published:** 2025-04-03

**Authors:** Tingting Zhang, Yinchen Yu, Jianyu Li, Li Zheng, Shiwei Chen, Jianjun Mao

**Affiliations:** 1 School of Advanced Manufacturing, Fuzhou University, Jinjiang 362251, China; zhangtt@fzu.edu.cn (T.Z.); q13062199310@163.com (Y.Y.); 15880434035@163.com (S.C.); 2 Jinjiang City Fuzhou University Science and Education Park Development Center, Fuzhou University, Jinjiang 362251, China; 3 Stockbridge School of Agriculture, University of Massachusetts Amherst, Amherst, MA 01003, USA; jianyuli@umass.edu; 4 Key Laboratory of Marine Eco-Environmental Science and Technology, First Institute of Oceanography, Ministry of Natural Resources, Qingdao 266061, China; zhengli@fio.org.cn; 5 State Key Laboratory for Biology of Plant Diseases and Insect Pests, Key Laboratory of Natural Enemy Insects, Ministry of Agriculture and Rural Affairs, Institute of Plant Protection, Chinese Academy of Agricultural Sciences, Beijing 100193, China

**Keywords:** transcriptome, nutritional, artificial diet, *H. axyridis*, juvenile hormone III

## Abstract

Artificial diets applied in the mass-rearing propagation of *H. axyridis* can improve reproductive ability by optimizing the feeding formula. This study used transcriptome data to investigate the effects of various artificial diets on the growth and development of *H. axyridis*. Results indicate that spawning increased with the low-fat and JH III-supplemented artificial diet (Diet 3). Furthermore, the highest glycogen content found in Diet 3 was significantly different from the other two groups. Triglyceride content decreased as adult feeding time increased in the three artificial diet groups, with the fastest decrease observed in the low-fat diet (Diet 2). Protein content increased gradually in the high-fat diet (Diet 1) group compared to the other treatment groups. The adults reared on low-fat artificial diets, when compared to those on artificial diets supplemented with juvenile hormones at the transcriptome level, were found to have upregulated genes enriched in ubiquitin-mediated proteolysis, ribosome biogenesis, and the hedgehog signaling pathway. In contrast, the genes upregulated in the latter group were enriched in oxidative phosphorylation, amino acid biosynthesis, and the metabolism of other amino acids. The results suggest that nutritional status significantly affects the growth and development of *H. axyridis* and has practical implications for the artificial feeding of natural pest enemies.

## Simple Summary

The *Harmonia axyridis* is an important natural enemy insect that has received widespread attention in research and the application of biological pest control. Currently, suitable artificial feed for the mass-rearing of the *H. axyridis* has not been found, which affects the application of this natural enemy in biological control. This study found that the egg production of artificial feed without added egg yolk is similar to that of artificial feed without added egg yolk supplemented with juvenile hormone III. Compared to the control artificial feed, the egg production significantly increased, which is of great significance for further exploring the effects of non-insect source artificial feed on the growth and development of the *H. axyridis*.

## 1. Introduction

*Harmonia axyridis*, as an important natural enemy, plays a crucial role in biological control. It exhibits strong stress resistance, high prey consumption, and a broad prey spectrum, primarily preying on agricultural pests such as aphids, coccids, lepidopteran eggs, and other insects [1,2]. With the development of global biological control, *H. axyridis* has been introduced into various countries in North America, South America, Europe, and Africa [3]. Currently, mass-rearing practices for *H. axyridis* rely on natural prey, making the process costly and labor-intensive [2]. Since the nutritional composition of insect-based sources shares the same insect origin as ladybirds’ natural food, their nutritional categories and contents are closer to natural diets. Consequently, insect-based diets have been more widely applied in ladybird artificial feeding systems. However, they present challenges including complex prey-rearing processes, higher costs, and difficulty in material acquisition. In contrast, non-insect-based artificial diets offer advantages such as easier material availability, lower costs, and more convenient management [4]. The mass-rearing propagation of *H. axyridis* remains an important issue that needs to be addressed. In Japan, artificial diets and drone pupal feeds were used to show that potassium ions and water-soluble substances from drone pupae promote the capacity of *H. axyridis* [5,6]. Zhang et al. compared the amino acid content of artificial diets and pea aphids for *H. axyridis* by measuring and adding three limiting amino acids to the diet using relative content calculations but found no significant effect [7]. This suggests that simply supplementing amino acids does not improve the feeding effect of artificial diets. We can only add amino acids that are lacking in the diet and cannot remove excess amino acids compared to natural prey, leading to an amino acid nutritional imbalance or nutritional overdose toxicity. Ricupero found that a mixture of moth eggs and brine shrimp cysts supported the rearing of *H. axyridis* [8]. Seko et al. revealed that the combination of *A. salina* cysts with sucrose positively influences the reproduction and biocontrol activity of flightless *H. axyridis* populations in greenhouse [9]. A liver-based artificial diet was tested, but *H. axyridis* reared on this diet did not develop successfully [10]. Specty et al. found that *E. kuehniella* eggs were nutritionally superior to *A. pisum* in terms of amino acid and fatty acid content and composition [11]. The expansion of natural enemy insects is affected by seasons, climate, and geography, resulting in limitations in stability and sustainability during the feeding process [12]. Non-insect-derived artificial diets have a wide range of inexpensive sources that can reduce the feeding costs of *H. axyridis*. However, non-insect-derived artificial diets can also directly affect the reproductive capacity of *H. axyridis*. For example, adult ladybirds fed egg yolk, beef liver, etc., do not lay eggs, but when the diet is changed to aphids, the female egg-laying ability was significantly improved [13].

In recent years, research on non-insect-derived artificial diets for *H. axyridis* has been limited, with persistent challenges in addressing developmental delays and reproductive degeneration. These unresolved issues significantly hinder the advancement of artificial diet formulations and impede the mass production of this species for biocontrol applications. The main components of non-insect-derived artificial feed for ladybugs are pork liver and yeast. Li et al. explored the effects of different diets on survival and reproduction [14]. They found that adding glucose and trehalose to non-insect-derived artificial feed significantly increased the egg-laying capacity of *H. axyridis*. Huang et al. demonstrated that different diets affect the abundance of gut microbiota and biological parameters such as the hatching time, developmental period, lifespan, pupal stage, and survival rate [15]. Zhang et al. added vitellogenin (Vg) to artificial feed and found that adding Vg improved the reproductive capacity of *H. axyridis* [16]. Some artificial feeds using eggs are rich in nutrients, such as fat and protein; egg yolks contain high cholesterol (12–15 mg/g) [17]. According to previous reports, the sterol components in insect feed are not crucial, and only a small amount of cholesterol in the feed supports the growth and development of insects. Regardless of their structure, cholesterol and other sterols are used for non-metabolic purposes [18]. Female fruit flies fed diets containing high protein levels had smaller ovaries and showed less active oogenesis. These studies show that female fruit flies fed diets containing high protein levels exhibit decreased fertilization rates and delayed offspring development [19].

The nutrient intake and hormones of insects are critical factors that determine their growth, development, and reproductive status. Among them, lipids are the main way to store energy [20]. Larval tissue formation in embryogenesis depends on maternally derived yolk proteins, while adult tissue growth during metamorphosis utilizes amino acids from hexamerins stored in the body fat before pupariation [21]. Glycogen, the primary carbohydrate reserve in Drosophila, serves as a critical metabolic safeguard [22]. Triacylglycerols (TAG) play an important role in the maintenance of energy homeostasis [23]. To date, eight different types of JHs have been identified in insects: JH 0, JH I, JH II, JH III, 4-methyl JH I, bis-epoxide JH III (JHB III), skipped bis-epoxide (JHSB III), and methyl farnesoate (MF). Methyl farnesoate, 10,11-epoxide (JH III) is the most common JH in insects [24]. JH III can promote oogenesis, but the removal of the allatectomy will eliminate this effect [25]. It regulates insect growth, development, and reproductive capacity [26]. Additionally, adding juvenile hormone analogs to the feed significantly shortened the pre-oviposition period of *Coccinella septempunctata*, although it reduced the survival rate and increased reproductive capacity [27].

We removed egg yolk from the artificial feed to reduce cholesterol, added juvenile hormone analogs [28], and examined the effects of different artificial feeds on the laying capacity of *H. axyridis*. We found that Diet 2 and Diet 3 improved the reproductive capacity compared to Diet 1. We also discovered genes related to reproductive regulation. The results of this study lay a foundation for enhancing ladybug reproduction and provide theoretical support for mass-rearing and biological control.

## 2. Materials and Methods

### 2.1. Insect Rearing

The *Harmonia axyridis* were raised in the laboratory of Jinjiang Campus of Fuzhou University. They were continuously fed on pea aphids for more than three generations in the laboratory. The pupae of the tested ladybugs were fed with three different artificial feeds after they emerged. The experimental adults were reared in artificial climate chambers at a temperature of 25 °C with a 16 h of light and 8 h of dark (16 L: 8 D) photoperiod in the laboratory at 65% ± 5% relative humidity. Three types of diets were tested in this study: (1) Diet 1: artificial diet to feed *H. axyridis*; (2) Diet 2: Diet 1 without egg yolks; (3) Diet 3: juvenile hormone III was added to Diet 2 (Aladdin, Shanghai, China). Artificial Diet 1 was formulated based on the protocol established by Lu et al. with slight modifications [29]. The artificial diet used to feed *H. axyridis* includes the main components of pork liver, eggs, sugar, and yeast. All diet preparations were conducted under laboratory conditions, where the formulated diets were portioned into aliquots and cryopreserved at −20 °C until further experimental application (Table 1).

### 2.2. Fecundity

The reproductive capacity of the *H. axyridis* adults was assessed under three different artificial diets. On the first day after emergence, female and male adults were paired and placed in individual boxes for rearing. They were provided with sufficient artificial diet daily, and observations were conducted every 24 h. Each treatment included 15 replicate pairs. The following reproductive indicators were recorded: pre-oviposition period, egg weight, hatching rate, and total egg production over 30 days. This study focuses on the direct impact of artificial feed on reproductive efficiency. Since the adult lifespan of *H. axyridis* is relatively long and oviposition occurs over an extended period, only the cumulative number of eggs laid within 30 days from the first oviposition was counted.

### 2.3. Dietary Effects on Ovarian Development

Females were randomly selected on day seven post-emergence. The ovaries were dissected in saline solution (125 mM NaCl, 5 mM KCl, and 1.85 mM CaCl_2_, pH 6.5) (Millipore Sigma, Burlington, MA, USA), disrupted with a needle, and observed at 20-fold magnification (SZX16-ILLT; Olympus, Tokyo, Japan). Three replicates were performed for each group.

### 2.4. Measurement of Energy Substances in Different Artificial Diets for H. axyridis

Adult female insects on days 0th, 10th, 20th, and 30th day of emergence were ground with liquid nitrogen. Samples (0.1 g) were ground, treated with PBS, centrifuged, and the supernatants were collected. The protein content in the supernatant was determined using the BCA method [30]. Finally, the absorbance of the solution was measured at 562 nm after the reaction ended to calculate protein content. Glycogen content was determined using the anthrone method [31]. The sample was digested in boiling sodium hydroxide, alkaline lysate deproteinization via perchloric acid, ethanol-mediated precipitation, ethanol washing, and anthrone reagent-based colorimetric quantification. Absorbance of the test solution was measured at 620 nm. The triglyceride (TAG) content was measured using Mukherjee and Mishra’s spectrophotometric assay [32]. The triglyceride (TAG) content was determined by adding 0.1 g of sample to a mixture of 1 mL of n-heptane and isopropanol (volume ratio 1:1), homogenizing it in an ice bath, centrifuging at 8000× *g* and 4 °C for ten minutes, taking the supernatant, and measuring the absorbance at 420 nm using a Multiscan SkyHigh spectrophotometer (Thermo Fisher Scientific, Waltham, MA, USA). Each reaction was performed in triplicate with three biological replicates.

### 2.5. Illumina Sequencing for Transcriptome Analysis

Total RNA was extracted from 7-day-old post-emergence female adults (3 adults per sample for every biological replicate) using EasyPure^®^ Simple Viral DNA/RNA Kit (Trans, Beijing, China) according to the manufacturer’s protocols, and then reverse transcribed and amplified using an Ovation Trio RNA Seq Library Preparation Kit (Trans, Beijing, China). The subsequent libraries were sequenced with an Illumina Novaseq platform at Novagene (Beijing, China). Raw sequencing data quality was evaluated using FastQC [33]. To obtain high-quality clean reads, technical artifacts in FASTQ files including adapter sequences, PCR primers, residual fragments, and bases with Phred scores ≤ 20 were trimmed using Cutadapt [34]. De novo transcriptome assembly was performed using Trinity software (http://TrinityRNASeq.sourceforge.net, accessed on 1 June 2024) with the filtered RNA-seq data [35]. Redundant contigs were clustered and removed via CD-HIT, generating a non-redundant unigene dataset. Differentially expressed genes (DEGs) were identified using the DESeq2 Bioconductor package, with thresholds set at |log2 Fold Change| ≥ 0 and a false discovery rate (FDR) < 0.05 [36]. Read data were submitted to NCBI SRA under accession PRJNA1053861.

The unigenes were searched by BLASTX against the non-redundant (Nr) protein database (http://www.ncbi.nlm.nih.gov/, accessed on 2 June 2024), Gene ontology (GO) (http://www.geneontology.org, accessed on 3 June 2024), Clusters of Orthologous Groups (COG) (http://www.ncbi.nlm.nih.gov/COG/, accessed on 6 June 2024), SwissProt (http://www.expasy.ch/sprot/, accessed on 7 June 2024), and the Kyoto Encyclopedia of Genes and Genomes (KEGG) (http://www.genome.jp/kegg/, accessed on 10 June 2024) database with a significant *p*-value less than 0.05. GO, COG, and KEGG enrichment analyses were performed on the significant DEGs by perl scripts in house.

### 2.6. Identification of Differentially Expressed Transcripts

Differential expression analyses of control and treatment groups were performed using the DESeq2 R package (version 1.20.0). DESeq2 provides statistical routines for determining differential expression in digital gene expression data using a model based on a negative binomial distribution. The resulting *p*-values were adjusted using Benjamini and Hochberg’s approach to control for the false discovery rate. Genes with an adjusted *p*-value ≤ 0.05 found by DESeq2 were assigned as differentially expressed.

### 2.7. Real-Time Quantitative PCR (qRT-PCR) Analysis

The primers for the target and reference genes were designed using the online Primer 3 program and are listed in Table 2. Actin was used as the internal reference gene. The qRT-PCR reaction volume of 20 µL contained 2 µL of cDNA template, 0.4 µL of forward and reversed primers, 10 µL 2× TransStart^®^ Green qPCR SuperMix, 0.4 µL of Passive Reference Dye (50×), and 6.8 µL of nuclease-free water (TransGen Biotech Co., Ltd., Beijing, China). The PCR reaction program was as follows: 94 °C for 30 s, followed by 45 cycles of denaturation at 94 °C for 5 s, 55 °C for 15 s, and an extension of 72 °C for 10 s. Real-time PCR was performed using an ABI Prims7500 system.

### 2.8. Statistical Analysis

The nonparametric Kruskal–Wallis’s test determined differences in net total eggs and pre-oviposition. The egg weights, hatching rates, protein glycogen, and triglyceride content results were determined using a one-way analysis of variance (ANOVA Least Significant Difference test). The mean values of replicates were expressed as mean ± standard error (SE, *n* = 3), and *p*-value ≤ 0.05 was considered statistically significant. Data were analyzed using SPSS software (version 16.0; SPSS, Chicago, IL, USA).

## 3. Results

### 3.1. Effects of Different Artificial Diets on H. axyridis Reproduction

The effects of the different artificial diets on the development and reproduction of *H. axyridis* were evaluated. Feeding different diets for 30 days resulted in different egg weights of 1.73, 1.83, and 1.77 mg, respectively, (*F* = 3; *df* = 2; *p* = 0.125) and different hatching rates at 83.33%, 85.00%, and 86.67%, respectively (*F* = 0.176; *df* = 2; *p* = 0.842). There were no significant effects on egg weight or hatchability of *H. axyridis*. During the 30-day oviposition period, our results indicate that the Diet 1, Diet 2, and Diet 3 females laid a total of 4.25, 29.05, and 24.65 eggs (average number of total eggs per female during the first 30 days), respectively. Total fecundity significantly differed between the Diet 2 and Diet 3 or Diet 1 group (*χ*^2^ = 7.486; *df* = 2; *p* = 0.024). Similarly, the pre-oviposition time of 12.78 and 10.90 days for Diet 2 and Diet 3 groups was significantly lower than that of Diet 1. There were significant effects on pre-oviposition time (the period from emergence to the first egg-laying) (*χ*^2^ *=* 5.588; *df =* 2; *p =* 0.061) (Table 2).

**Table 2:** Developmental and reproduction characteristics of *H. axyridis* fed on different diets.

Group	Total Egg	Egg Weight/mg	Hatchability/%	Pre-Oviposition/Days
Diet 1	4.25 ± 2.24 b	1.73 ± 0.03 a	83.33 ± 0.36 a	19.75 ± 3.68 a
Diet 2	23.58 ± 8.73 a	1.83 ± 0.03 a	85.00 ± 0.24 a	13 ± 1.17 b
Diet 3	24.65 ± 6.8 a	1.77 ± 0.03 a	86.67 ± 0.36 a	11 ± 0.69 b

Means ± SE of net total egg and pre-oviposition followed by different letters within a column indicate significant differences determined by the Kruskal–Wallis test (*p* < 0.05). The table’s egg weight and hatching rates are expressed as mean ± SE; the same letters within a column indicate no significant differences (One-way ANOVA test, *p* < 0.05).

### 3.2. Arrest of Ovarian Development After Feeding Different Artificial Diets

**Figure 1:**
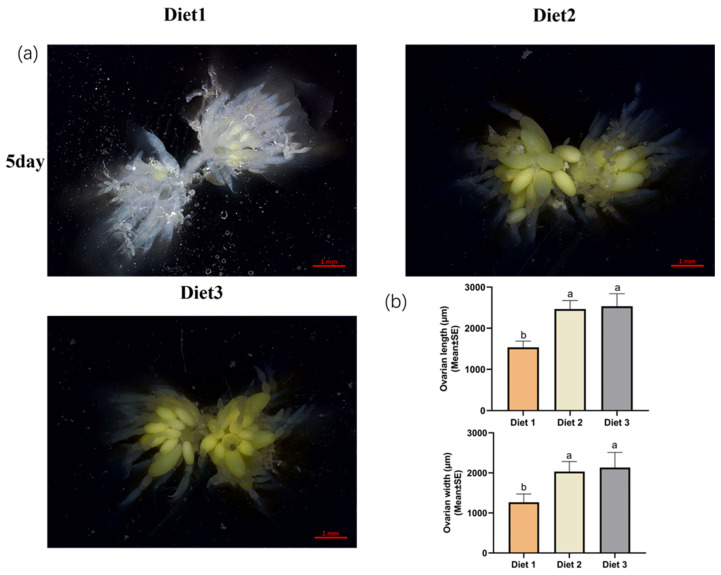
Effect of ovarian development of different artificial diets in *H. axyridis*. (**a**) Dissected ovaries five days after emergence. Diet 1 (standard, artificial), Diet 2 (low fat artificial), and Diet 3 (addition of juvenile hormone III to Diet 2). (**b**) ovarian length and ovarian width. The scale bar represents 1 mm. Different letters above each bar indicate significant differences among different treatments using One-way ANOVA test (*p* < 0.05).

Mature ovaries did not significantly differ between Diet 2 and Diet 3 insects. Adult females that emerged from different artificial diets were dissected five days post-eclosion. Diet 2 and Diet 3 groups showed more mature ovaries, egg chambers, and larger ovary scales than Diet 1 (Figure 1).

### 3.3. Effects of the Artificial Diets on Protein, Glycogen, and Triglyceride Content

**Figure 2:**
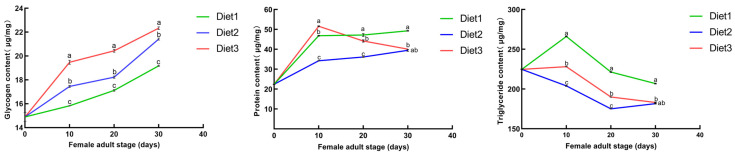
Protein, glycogen, and triglyceride content during the female adult stage after different artificial diets. Diet 1, Diet 2, and Diet 3 were fed on the first day of emergence. Protein, glycogen, and triglyceride contents were monitored at 0, 10, 20, 30, and 40 days of age. SPSS analyzed the significance of the differences in the nutrient content. The nutrient contents are shown as the means ± SEs and were found to be significantly different. Different letters above each bar indicate significant differences among different treatments using One-way ANOVA (*p* < 0.001) and Kruskal-Wallis test (*p* < 0.05).

The effects of artificial diets on protein, glycogen, and triglyceride contents were determined. We found no significant difference in the protein content of adult females on the 1st day of emergence. As the feeding time increased, the protein content in the Diet 3 group was significantly higher than that in the Diet 1 and Diet 2 groups on the 10th (*F* = 9218.847; *df* = 2; *p* < 0.001), 20th (*F* = 369.016; *df* = 2; *p* < 0.001), and 30th days (*χ*^2^ = 7.261; *df* = 2; *p = 0.027*) after emergence. The glycogen content on the 10th day (*F* = 831.353; *df* = 2; *p* < 0.001), 20th day (*F* = 942.361; *df* = 2; *p* < 0.001), and 30th day (*F* = 1165.412; *df* = 2; *p* < 0.001) were significantly affected by the different artificial diets. There were significant differences in triglyceride content on the 10th day (*F* = 60,867.206; *df* = 2; *p* < 0.001), 20th day (*F* = 16,504.961; *df* = 2; *p* < 0.001) and 30th day (*χ*^2^ = 7.322; *df* = 2; *p* = 0.026) (Figure 2).

### 3.4. Transcriptome Assembly and Differentially Expressed Genes

Libraries of control samples (Diet1-1, Diet1-2, and Diet1-3), libraries treated with sample 1 (Diet2-1, Diet2-2, Diet2-3), and sample 2 (Diet3-1, Diet3-2, and Diet3-3) were collected (only nine samples were analyzed by differential expression genes, DEGs). Three biological replicates were used for each sample. We obtained nine libraries containing clean reads (Diet1-1:42099192; Diet1-2:41807246; Diet1-3:42138400; Diet2-1:41918970; Diet2-2:44428656; Diet2-3:42094054; Diet3-1:44049024; Diet3-2:43360852; Diet3-3:44010148), generated for analysis after cleaning and checking. The percentage of GC content was analyzed in all nine libraries (Diet1-1: 39.36; Diet1-2: 38.19; Diet1-3: 39.28; Diet2-1: 39.26; Diet2-2: 39.02; Diet2-3: 38.06; Diet3-1: 39.21; Diet3-2: 38.69; Diet3-3: 38.52). The nine libraries of percentage ≥ Q30 were 94.17, 93.28, 93.95, 94.45, 93.75, 94.17, 93.95, 94.29, and 93.74%, respectively (Table 3).

**Figure 3:**
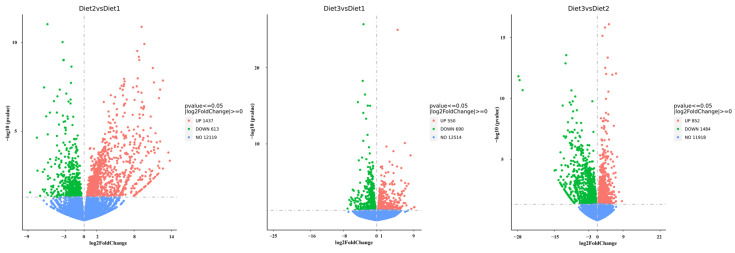
Scatter diagrams representing the comparisons of the genome-wide expression profiles of control and treatment. Note: Each green point represents a decrease, each red point represents an increase, and each blue point represents no significant difference in gene expression.

To explore the effects of different artificial feeds on the molecular mechanisms of *H. axyridis,* we used |log2 Fold Change| ≥ 0 and false discovery rate (FDR)-adjusted *p* value ≤ 0.05 to detect the differentially expressed genes (DEGs) in each group. Comparing Diet 2 with Diet 1, there were 2050 differentially expressed genes in the transcriptome, of which 1437 were upregulated and 613 were downregulated in Diet 2. Comparing Diet 3 with Diet 1, there were 1240 differentially expressed genes in the transcriptome, of which 550 were upregulated and 690 were downregulated in Diet 3. Comparing Diet 3 to Diet 2, there were 2336 differentially expressed genes in the transcriptome, of which 852 were upregulated, and 1484 were downregulated in Diet 3 (Figure 3).

### 3.5. GO Functional Annotation and KEGG Enrichment Analysis of DEGs

**Figure 4:**
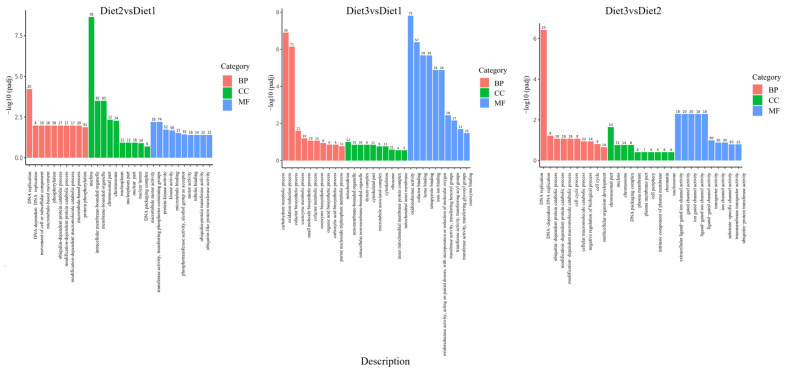
Summary of the annotations of differentially expressed genes in *H. axyridis*. The GO classifications are shown according to involvement in biological processes, cellular components, and molecular functions.

GO enrichment analysis was performed on DEGs, and the GO terms were screened with *p* value ≤ 0.05. Comparing Diet 2 with Diet 1, 901 GO terms were successfully annotated, including 243 biological processes, 331 cellular components, and 327 molecular functions. In the molecular function category, the DEGs were mainly enriched in the structural constituents of the cuticle, ion channel activity, and substrate-specific channel activity. Comparing Diet 3 to Diet 1, 648 GOs were annotated, including 183 biological processes, 146 cellular components, and 319 molecular functions. These genes were mainly enriched in the oxidation-reduction process, cofactor binding, and oxidoreductase activity. Comparing Diet 3 to Diet 2, 642 GOs were annotated, including 154 biological processes, 149 cellular components, and 339 molecular functions. DEGs were mainly enriched in transmembrane transporter activity, responses to stimuli, and transmembrane transport (Figure 4).

**Figure 5:**
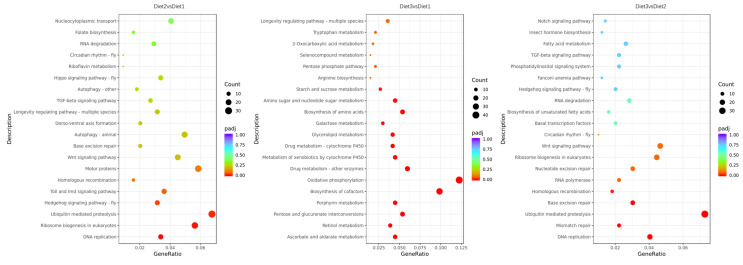
The 20 most significant KEGG pathways were selected from the KEGG enrichment results. In the figure, the abscissa indicates the ratio of differential genes annotated to the KEGG pathways to the total number of such genes; the ordinate stands for the KEGG pathways. The size of the points represents the number of genes annotated to the KEGG pathways, and the color from red to purple represents the significance level of enrichment.

The results showed the function of DEGs in different artificial diets using KEGG pathway analysis. In addition, comparing Diet 2 to Diet 1 in the KEGG pathway also included “DNA replication”, “ribosome biogenesis in eukaryotes”, “ubiquitin-mediated proteolysis”, and the “Toll and Imd signaling pathway”. Comparing Diet 3 to Diet 1, the enrichment KEGG pathway showed “biosynthesis of cofactors”, “oxidative phosphorylation”, “drug metabolism-other enzymes”, and “glycerolipid metabolism”. Comparing Diet 3 to Diet 2, the enrichment KEGG pathway involved “insect hormone biosynthesis”, “fatty acid metabolism”, and the “phosphatidylinositol signaling system” (Figure 5).

### 3.6. Identification of Development-Related Genes Differentially Expressed Under Different Artificial Diets

**Figure 6:**
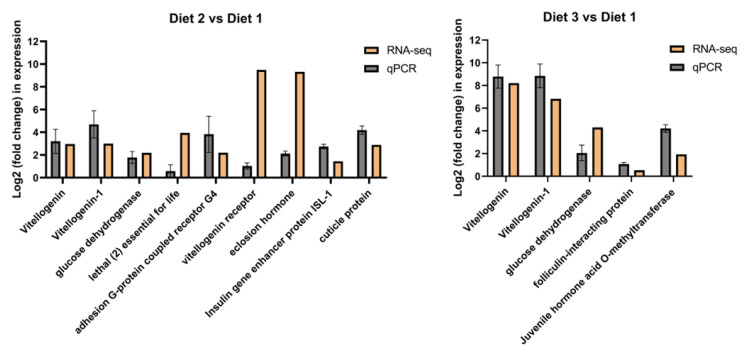
Analysis of differentially expressed genes under different artificial diets in *H. axyridis*. The expression changes for each candidate gene as measured by RNA-seq and qRT-PCR.

To validate the DEGs determined from the transcriptome results, reproduction- and nutrition-related genes were selected from the qRT-PCR analysis results. The genes and primers used for qRT-PCR are listed in Table 3. The expression trends of the 11 genes were consistent with the transcriptome sequencing results (Figure 6), indicating the high reliability of the RNA-seq results. Thus, qRT-PCR analysis confirmed the directional change in gene expression detected in the previous DEG analysis (Table 4).

## 4. Discussion

Natural prey is considered the optimal food source for the growth and development of lady beetles. However, its application in mass rearing is constrained by high costs, operational complexity, and dependency on host plant availability. Artificial diets, in contrast, circumvent these limitations. Nevertheless, studies have demonstrated suboptimal reproductive outcomes in lady beetles fed artificial diets. Chen reported that the seven-spotted lady beetle (*Coccinella septempunctata*) fed artificial diets exhibited a reproductive diapause-like state, characterized by delayed development of reproductive cells [37]. Additionally, their consumption rates were only one-fifth of those fed aphids. Similarly, Zhang found that the daily egg production of the striped lady beetle (*Propylea japonica*) reared on a pork liver-based artificial diet was significantly lower than that of the aphid-fed group [38]. Although artificial diets significantly prolonged adult longevity compared to aphid-fed groups, they resulted in markedly reduced fecundity [7].

The present study compared the effects of different non-insect artificial diets on the growth and development of *H. axyridis*. The primary nutritional component of egg yolk is fat. Fat and cholesterol mutually influence each other during metabolic processes [39]. For instance, when fat intake is excessive, fat may be transformed into cholesterol through a series of biochemical reactions, thereby causing an increase in cholesterol levels within the body of insects [40]. However, the effects of cholesterol on insect reproduction are not always linear. Excessively high cholesterol concentrations may exert an inhibitory effect on insect reproduction [41]. Previous studies have shown that high concentrations of cholesterol interfere with the normal synthesis and metabolic processes of hormones in insects, leading to abnormal development or functional impairment of the reproductive organs [41]. This study revealed that the total number of eggs in the low-fat diet was significantly higher than in the high-fat diet. Feeding a low-fat diet and juvenile hormone III accelerated ovary maturation and elevated reproductive performance in previtellogenic females, suggesting that vitellogenesis plays a crucial role in the reproductive regulation of *H. axyridis*. In *Blatella germanica*, JH regulates reproductive processes, including previtellogenic development, vitellogenesis, and oogenesis [42]. Li et al. demonstrated that the solid reproductive ability of *A. craccivora* was closely related to JH III [43]. Our results indicate that an artificial diet with egg yolk removal can promote egg laying, and an artificial diet supplemented with juvenile hormones can also increase fecundity in adults; however, there was no significant difference compared with the egg yolk removal group (Table 2).

Comparative transcriptomic analysis revealed that some DEGs affected the reproductive ability of *H. axyridis*. When annotating the DEGs, we focused on the expression of genes related to development and reproduction. Comparing Diet 2 with Diet 1, we first found upregulation of the insulin gene enhancer protein ISL-1 (Islet-1), cuticle protein, vitellogenin, eclosion hormone (EH), and Lethal (2) essential for life [l(2)efl] and vitellogenin receptors, which participate in development. Islet-1 is important in developing and forming various organs and tissues [44]. The insect exoskeleton (cuticle) is composed of chitin and cuticle proteins. Recently, cuticle proteins have been attractive models for studying time- and tissue-specific cell differentiation mechanisms during animal development [45]. Cuticle protein genes are involved in insect development and cuticle formation during ecdysis [46]. It is essential to take up vitellogenin and dominate ovary maturation in insects. This study observed increased expression of the vitellogenin receptor (VgR) [47]. VgR is essential in Vg uptake, enhancing oocyte maturation and insect fecundity [48]. The total number of eggs spawned increased in Diet 2. Jing et al. found that JH stimulates VgR phosphorylation and that phosphorylated VgR migrates from the oocyte cytoplasm to the cell membrane, where it binds to Vg and undergoes VgR/Vg endocytosis [49]. Its role is crucial in regulating vitellogenin metabolism in oocytes. This process is essential for oocyte development and maturation and contributes to successful reproduction in various species. Elevated Vg and VgR expression levels may have important implications for understanding reproductive physiology and identifying potential targets for further research. EH, a neurohormone critical for modulating insect pre-ecdysis behavior, orchestrates behavioral transitions during the terminal phase of each molting cycle [50]. Research has found that *l(2)efl* in *D. melanogaster* are continuously expressed during development. While the mechanism is unclear, this *l(2)efl* homolog induces phosphorylation of eukaryotic initiation factor 2α (eIF2α), thereby inhibiting protein biosynthesis [51].

Comparing Diet 3 with Diet 1, our transcriptome analysis revealed a significant increase in Vg, juvenile hormone acid O-methyltransferase, glucose dehydrogenase (GLD), folliculin-interacting protein (FNIP), and G protein-coupled receptor G4 (ADGRG4) expression in Diet 3, crucial for egg development and reproduction in many species. Vitellogenin (Vg), a key precursor of the yolk protein vitellin (Vn), functions as a vital energy reserve in oviparous species [16]. Vgs are synthesized predominantly in body fat cells in a tissue-, sex-, and stage-specific manner. These proteins are then secreted into the hemolymph and internalized by developing oocytes through receptor-mediated endocytosis [52]. Vg affects oocyte maturation, egg formation, and insect embryonic development [53]. In addition, juvenile hormone acid O-methyltransferase (JHAMT) has been suggested as a crucial enzyme in the JH-branch, regulating juvenile hormone synthesis [54]. JH is a gonadotropin that induces the synthesis of yolk proteins in body fat and promotes ovarian development [55]. The upregulation of GLD observed in our transcriptome data is consistent with findings in honeybees, where increased GLD activity was shown to be significantly correlated with ovarian development [56]. This conserved mechanism suggests that the artificial diet may enhance reproductive efficiency through similar nutrient-sensing pathways. FNIP1, as a substrate of AMPK, regulates the translocation of TFEB to the nucleus, thereby enhancing lysosomal and mitochondrial biogenesis under metabolic stress conditions [57]. *He6* has demonstrated an essential role of this AGPCR in spermatogenesis and fertility [58].

## 5. Conclusions

The work presented here showed that removing egg yolks from an artificial diet increased egg production in *H. axyridis*. In addition, adding JH III to Diet 2 also increased egg production compared to Diet 1, but there was no difference in egg production between Diet 2 and Diet 3 groups. Therefore, low-fat artificial feed is suitable for rearing *H. axyridis*. Feed formulation optimization and efficiency enhancement will be required to fulfill the requirements for the mass-rearing, artificial breeding, and biological control of *H. axyridis*. Although this paper confirmed that low-fat artificial diets can effectively support the rearing of adult *H. axyridis* through studying the effects of different artificial diets on the growth and development of *H. axyridis*, it should be particularly noted that the current study did not involve functional verification experiments of key candidate genes (such as Vg and FNIP) (e.g., RNA interference (RNAi) technology). This limitation stems from the fact that such studies usually focus on the macroscopic effect assessment of artificial diets on insect phenotypes (such as growth and development, reproductive performance), rather than the analysis of their molecular regulatory mechanisms. Based on this, subsequent studies will systematically analyze the regulatory network of nutrition signal pathway-related genes such as Vg and FNIP in the nutrition metabolism of *H. axyridis*, especially their dynamic response mechanisms to artificial diet components. Such functional verification studies are of crucial significance for transforming transcriptomic discoveries into targeted feed optimization strategies and will also provide theoretical support for establishing a precise feeding system based on molecular mechanisms.

## Figures and Tables

**Table 1:** The percentage composition of each component per 100 g of artificial diets.

Ingridients	Diet 1	Diet 2	Diet 3
Fresh pig liver	35%	35%	35%
Whole egg	30%	-	-
Egg white	-	30%	30%
Brown sugar	14.9%	14.9%	14.9%
Pork	9.9%	9.9%	9.9%
Yeast extract	5%	5%	5%
Vitamin C	0.2%	0.2%	0.2%
Linseed oil	5%	5%	5%
Juvenile hormone III	-	-	10%

**Table 3:** Illumina-sequencing data analysis results.

Sample	Library	Raw Reads	Raw Bases	Clean Reads	Clean Bases	Error Rate	Q30	GC pct
Diet1-1	FRAS230248041-1r	43092126	6.46G	42099192	6.31G	0.02	94.17	39.36
Diet1-2	FRAS230248044-1r	42727980	6.41G	41807246	6.27G	0.03	93.28	38.19
Diet1-3	FRAS230248050-1r	43068620	6.46G	42138400	6.32G	0.03	93.95	39.28
Diet2-1	FRAS230248053-1r	43065702	6.46G	41918970	6.29G	0.02	94.45	39.26
Diet2-2	FRAS230248056-1r	45325406	6.8G	44428656	6.66G	0.03	93.75	39.02
Diet2-3	FRAS230248059-1r	43014486	6.45G	42094054	6.31G	0.02	94.17	38.06
Diet3-1	FRAS230248068-1r	44711108	6.71G	44049024	6.61G	0.03	93.95	39.21
Diet3-2	FRAS230248071-1r	44141014	6.62G	43360852	6.5G	0.02	94.29	38.69
Diet3-3	FRAS230248074-1r	45319418	6.8G	44010148	6.6G	0.03	93.74	38.52

**Table 4:** Information of *H. axyridis* cultivable bacteria by Blast against GenBank database.

Gene ID	Homologous Function in Nr	Primer (5′-3′)	Expression Level	Fragment Size (bp)	Tm (°C)
123686364	Vitellogenin-1	GATGGATACGAGTACCCACT CTTTGCTTAGCCAAGACG	up	149	58
123686370	Vitellogenin	AAGAGTCGCGCACAGAAGAA TGCAATGGGACTTGCAAACG	up	199	59
123674747	Glucose dehydrogenase	ACCCTTCGTGATGGTCTG CCTGTAGCCTGATAAGTTTC	up	137	59
123677096	Adhesion G-protein coupled receptor G4	AAGTGTAAGTTAGTAGGGTGTT TAGGAATAAGTGCGAAAC	up	172	59
123689048	Vitellogenin receptor	AATCACCCAGAACGTCACCC CCGTCAGGCTGGACAGATTT	up	186	58
123676458	Eclosion hormone	TTGAAGCCTATAAGACTGG CAAGAATGGTGCAACTGA	up	185	57
123678499	Folliculin-interacting protein	GGCTACCTGTTTGCCTCG GGATGTCCTACCGCACCA	up	111	58
123678119	Lethal (2) essential for life	ATTTCCAGGCAGTTTGTT GGGATGGTTTTATGATCAATACTCT	up	154	59
123672779	Cuticle protein	CGCGTTCTCCTACAAAGTGG CGCTCCGACATTACTCCTCT	up	226	58
123688747	Insulin gene enhancer protein ISL-1	ATGGGGCGATGCAAGGTATA GCCGTGCATCTGGTTAACAA	up	191	59
123684308	Juvenile hormone acid O-methyltransferase	TCAGGAGATGGACACACTCTG ACTCTGCTTGACAACCCAGT	up	240	58
123674045	β-actin	ACCCATCTACGAAGGTTATGC CGGTGGTGGTGAAAGAGTAA	up	122	57

## Data Availability

The raw reads of the transcriptome sequencing were deposited in NCBI under the project of PRJNA1191902.
